# Evaluation of efficacy and safety of intradermal delivery of vaccines through microneedle(s) in human beings: a protocol for a systematic review

**DOI:** 10.1186/s13643-022-02046-8

**Published:** 2022-08-13

**Authors:** Ishumeet Kaur Bajwa, Navneet Kaur, Jeanne M. Dsouza, Joseph L. Mathew

**Affiliations:** 1 Department of Pediatrics, Post Graduate Institute of Medical Education and Research, Chandigarh, 160012 India; 2 Kasturba Medical College, Manipal University, Manipal, 576104 India

**Keywords:** Microneedle, Vaccine, Intradermal, Human, Safety, Efficacy

## Abstract

**Background:**

Microneedles are defined as micron-sized projections with an insertion length ranging from 20 to 1500 μm and an external diameter up to 300 μm. Medications administered through microneedles diffuse through the deeper layers of the skin, into the systemic circulation, with minimal stimulation of pain-sensitive nerve endings. The rich presence of dendritic cells in the dermis makes microneedle-based vaccine delivery an attractive option. This systematic review will evaluate the efficacy and safety of intradermal delivery of vaccines using microneedles, in human beings.

**Methods:**

We will search the following databases for studies reporting the efficacy and/or safety of intradermal delivery of vaccines using microneedles: Epistemonikos and the Cochrane Library for systematic reviews and MEDLINE (through PubMed), EMBASE, Cochrane CENTRAL, LIVIVO, Web of Science, Scopus and CINAHL databases for primary studies. We will also search grey literature databases and hand search reference lists of relevant studies. We will include randomised and quasi-randomised trials in human beings (any age), using microneedles (any material, length or bore) to deliver vaccines intradermally, wherein outcomes reflecting efficacy, safety, pain responses, participant satisfaction or cost are reported. We will additionally include non-randomised observational studies for long-term safety outcomes that are not reported in trials. Eligibility for inclusion will be independently determined by two reviewers. The risk of bias of the included studies will be assessed using the Cochrane RoB2 Tool (for randomised trials) and Newcastle–Ottawa Scale (for other study designs). Data on efficacy and safety will be pooled through meta-analysis (where feasible). We will explore the heterogeneity amongst randomised trials, using the Higgins and Thompson *I*^2^ method. We will undertake sensitivity analysis to explore the impact of study quality and subgroup analysis based on the age of participants, length of microneedle and vaccine dosage. The GRADE approach will be used to estimate the confidence in the evidence.

**Results:**

This is a protocol for a systematic review; hence, there are no results at this stage.

**Discussion:**

The proposed systematic review will provide evidence on efficacy, safety, pain responses, participant acceptability and cost in human beings (adults and children) for vaccines administered through the intradermal route using microneedles. Since intradermal injections using microneedles are associated with less pain due to their short lengths and narrow bores, we anticipate that delivery of vaccine antigens using this method could be a safe, efficacious and less painful alternative compared with conventional injections using hypodermic needles. The evidence in this review will be useful for policymakers, vaccine manufacturers and healthcare providers to consider this approach for the vaccination of infants and children in routine immunisation programmes. Therefore, we plan to disseminate the review through a peer-reviewed journal publication and will also provide data that cannot be included in the published version to anyone upon reasonable request.

**Systematic review registration:**

PROSPERO CRD42020213608

**Supplementary Information:**

The online version contains supplementary material available at 10.1186/s13643-022-02046-8.

## Introduction

Microneedles are defined as micron-sized projections with lengths ranging from 20 to 1500 μm and external diameter less than 300 μm [1]. Application of microneedles to the skin, creates microscopic pores, allowing medications or vaccines to penetrate across the stratum corneum layer, into the dermis or subcutaneous tissue, and thence into the systemic circulation. The dermis possesses a rich supply of dendritic cells that are efficient antigen-presenting cells, capable of initiating the cascade of immunogenic responses leading to antibody production. Intradermal injections using microneedles are associated with less pain (compared to conventional hypodermic needles) because they have very short lengths and narrow bores, thereby minimising the stimulation of pain-sensitive nerve endings in the deeper part of the dermis and subcutaneous layer [2]. Therefore, intradermal delivery of vaccine antigens could be a safe, efficacious and less painful alternative compared with conventional injections using hypodermic needles.

A few studies using different designs, methods and outcome parameters have explored the safety and efficacy of using microneedles to deliver vaccines in various animal models. The vaccines include dengue vaccine in mice [3]; hepatitis B vaccine in rhesus macaque, mice and pig [4–6]; influenza vaccine in mice [7, 8]; hepatitis C vaccine in pigs [9]; respiratory syncytial virus (RSV) vaccine in mice [10]; diphtheria toxoid in mice [11, 12]; Ebola vaccine in mice [13, 14]; measles-rubella (MR) vaccine in macaques [15]; tetanus toxoid (TT) in mice [16]; rotavirus vaccine in pig [17]; measles vaccine in macaque [18]; and inactivated polio vaccine (IPV) in macaque [19].

In human beings, Bacille Calmette-Guerin (BCG) vaccine and purified protein derivative (PPD) used for tuberculin testing have been administered intra-dermally for decades but using conventional hypodermic needles. More recently, inactivated polio vaccine has also been administered intradermally in a dose-sparing effort (compared to the usual intramuscular route). However, intradermal vaccine administration through microneedles has not been widely studied for these applications. Very recently, tuberculin-purified protein derivative administration has been attempted using microneedles [20]. Some studies explored the administration of influenza vaccine through microneedle array patches in healthy volunteers [21–23] and anti-rabies vaccine in healthy volunteers [24] and suggested that the approach is feasible.

Since microneedle-based intradermal vaccine delivery could be a safe, efficacious, effective and economic method for vaccination, there is a need for a well-designed systematic review to examine the safety and efficacy of using microneedles for vaccine administration in humans.

## Objective

The objective of this systematic review is to identify, appraise and synthesise research evidence on the intradermal delivery of vaccines using microneedles, in human beings. This will allow a better understanding of the potential for using microneedles for intradermal delivery of vaccines in routine immunisation programmes across the world.

The specific review question is: What is the efficacy and safety of intradermal delivery of vaccines using microneedles in human beings?

## Methods

### Prospero registration and PRISMA-P statement

**Fig. 1:**
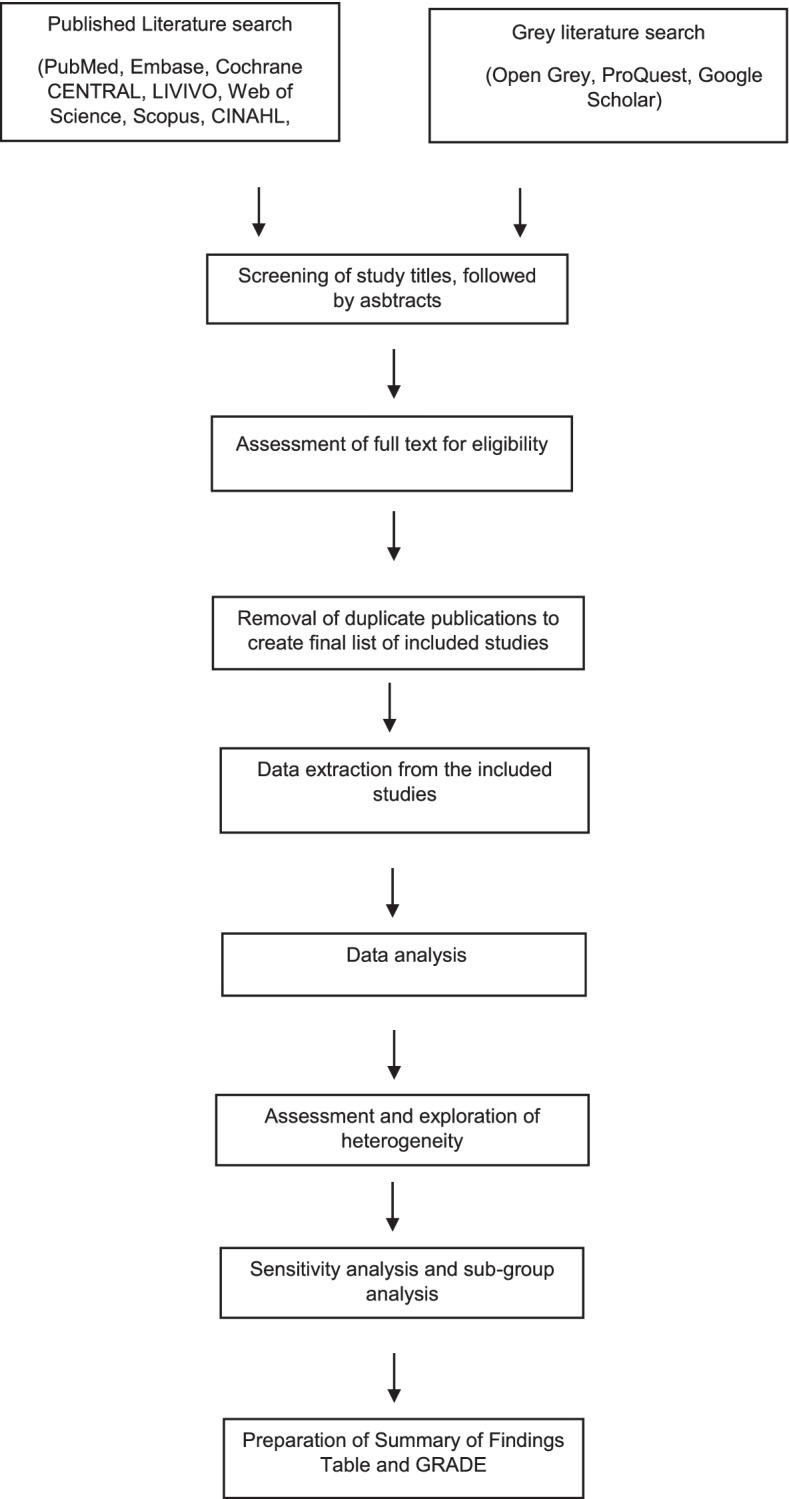
Flowchart of the systematic review

This protocol has been registered within the PROSPERO database (CRD42020213608) and will follow the relevant domains of the Preferred Reporting Items for Systematic Reviews and Meta-Analyses-Protocols (PRISMA-P) statement [25–27]. Figure 1 summarises the flow of the systematic review process.

### Criteria for considering studies for this review

#### Types of studies

We will include randomised controlled trials (RCTs) and quasi-randomised trials to evaluate the (a) efficacy of microneedle-administered vaccines and (b) expected short-term local and systemic reactogenicity events from microneedle-administered vaccines (e.g. erythema, tenderness, pruritis, fever, myalgia).

Additionally, we will include non-randomised (observational) studies for the purposes of addressing unexpected, rare or delayed adverse events that are unlikely to be reported in randomised trials due to trial size, trial duration or restricted eligibility criteria for participants in randomised trials.

We will exclude studies related to animal experiments, in vitro experiments and ex vivo human studies.

#### Types of participants

We will include studies that involve human participants (irrespective of age, gender, disease or health condition) to evaluate the safety and efficacy of intradermal delivery of vaccines using microneedles.

We will exclude studies done on human cadavers and post-mortem studies.

#### Types of intervention

We will include studies where microneedles (any material, any length, any diameter, any type) are used to administer vaccines intradermally (any vaccine, any type, any dosage, any number of doses).

We will exclude studies using microneedles, if they deliver vaccines at non-intradermal sites, for example, subcutaneous injection.

#### Types of comparison

We will include studies reporting the following types of comparison:

- The same vaccine (as the intervention arm) delivered using a conventional hypodermic needle into any site (such as intramuscular, subcutaneous, or intradermal)
- The same vaccine (as the intervention arm) delivered using conventional non-injectable routes for the specific vaccine (such as oral or intranasal)
- The same vaccine (as the intervention arm) delivered using jet injectors
- Placebo administered using microneedles or microneedle patches
- Head-to-head comparison of the same vaccine delivered with different types of microneedles (such as different lengths, diameters, material, geometry), with or without an arm/group in the trial receiving one of the comparisons listed in (a) to (d) above

#### Types of outcome measures

The following are the primary outcome:

1. Protective efficacy, i.e. post-vaccination incidence of the vaccine-preventable disease.
2. Seroprotection defined as follows:
  - For vaccine-preventable diseases with a widely accepted immune correlate of protection (ICP) or where there is a defined threshold value for a specific immune parameter that is accepted as indicating vaccine-induced protection from disease, seroprotection rate will be defined as the percentage of vaccinated persons with an immune response above the defined ICP or threshold value.
  - For vaccine-preventable diseases without an ICP or threshold values, seroprotection will be defined as the percentage of vaccinated persons with at least a four-fold rise in antibody titres from pre- to post-vaccination.

The following are the secondary outcomes:

1. Efficacy outcomes:
  - Other surrogate measures of vaccine efficacy or correlates of vaccine-induced immunity including, but not limited to, geometric mean antibody concentrations (GMCs), geometric mean antibody titres (GMTs), pre- to post-vaccination geometric mean ratio (GMR), measurements of functional antibody and/or binding antibody or long-term measurements of efficacy
2. Pain response (recorded using any validated tool) during or immediately after vaccination
3. Safety outcomes:
  - Incidence of local adverse reactions
  - Incidence of systemic adverse reactions
  - Incidence of serious adverse events.
4. Other outcomes:
  - Participant satisfaction
  - Participant acceptability
  - Cost of vaccination

### Search methods for identification of studies

#### Information sources

Two authors will independently undertake a literature search through the following electronic databases: Epistemonikos and the Cochrane Library for systematic reviews; and MEDLINE (through Pubmed), EMBASE, Cochrane CENTRAL, LIVIVO, Web of Science, Scopus and CINAHL databases for primary studies. All searches will be run from inception to 30 September 2021, or the actual date of publication of the protocol, whichever is later. There will be no restrictions based on language or geographies.

We will conduct literature searches using combinations of MeSH terms and synonyms of the following keywords and their variations: microneedle, vaccine and intradermal. A typical search strategy in PubMed is shown in Additional file 1. Pilot testing using this strategy yielded approximately 6000 results in PubMed alone, suggesting that the strategy is robust and unlikely to miss eligible studies.

#### Additional searches

##### Handsearching

We will hand-search reference lists of all primary studies and review articles to identify additional studies.

##### Grey literature

We will conduct a grey literature search to identify studies not indexed in the databases listed above, using OpenGrey, Proquest and Google Scholar.

We will use the Peer Review of Electronic Search Strategies (PRESS) checklist for systematic reviews and for structured reviews of our literature search strategies. The checklist is designed to identify errors in the search strategy and enhance the search.

### Study records

#### Data collection, management and synthesis

##### Selection of studies

We will follow a step-wise approach to identify studies eligible for inclusion in this systematic review. Two review authors will independently screen the titles, followed by the abstracts of studies identified through the searches, in order to determine eligibility. We will then screen the full-text study reports/publications of the potentially eligible studies, as well as those where no abstract is available. Thereafter, two authors will independently examine the full-text versions in detail and identify studies for inclusion, and record reasons for exclusion of the ineligible studies. Any disagreements will be discussed and resolved amongst the review authors, with arbitration by an external expert if necessary. A table of excluded studies will be presented with reasons for exclusion.

After the elimination of duplicate publications, a final list of included studies will be prepared. A PRISMA flow diagram will be created to illustrate the search results and the process of screening and including studies. The study screening form as well as data extraction form to be used in this systematic review will be pilot tested in advance to ensure there are no errors.

##### Translation of languages other than English

Publications in languages other than English will be subject to initial translation of the abstract using open source software. If this indicates potential inclusion, or if the translation is inadequate to permit a decision, an attempt will be made to obtain a formal translation of the full text. If this cannot be done, the authors will categorise the study as ‘awaiting classification’ to ensure transparency in the review process.

#### Data management

We will use Rayyan (https://www.rayyan.ai) for the management of the screening and data extraction stages of the systematic review.

#### Data collection process

Two review authors will independently extract the following information from each included study. Any disagreements will be discussed and resolved amongst review authors, with arbitration by the senior author.

1. Study characteristics including study design, duration of study, number of study sites, study context (setting, location) and date of publication.
2. Participants: inclusion criteria, exclusion criteria, age, gender, healthy or disease state, severity of condition and sample size.
3. Interventions: type of microneedle used, microneedle material, microneedle length, microneedle bore, presence of bevel, vaccine used, vaccine dosage, number of vaccine doses administered, criteria for evaluating efficacy, criteria for evaluating safety, and tools used to measure pain response(s).
4. Comparisons: type of needle used viz. conventional hypodermic needle (length, bore, design), jet injector(s) or placebo delivered by microneedles or microneedle patches. Route of administration of vaccines (intra-muscular, subcutaneous, intradermal, oral, intranasal). In studies with comparison groups, the same data as for the intervention group will be extracted.
5. Outcomes: we will extract data on the outcomes listed previously, from each trial (including the definitions of outcomes used by trial authors, outcome assessor(s), tools/instruments/methods used, units of measurement where appropriate, upper and lower limits for any scales used and the time-point(s) of outcome measurement).

#### Dealing with missing data

We will contact the corresponding authors of studies where data is/are missing and try to obtain the missing data. In instances where missing outcome data can reasonably be assumed to be ‘missing at random’, we will analyse only the available data (i.e. conduct an ‘available case analysis’).

For missing data in dichotomous outcomes, we will conduct additional analyses by assuming (a) favourable outcomes in all those with missing data, (b) unfavourable outcomes in all those with missing data, (c) best case scenario (i.e. favourable outcomes in all those with missing data in the intervention arm and unfavourable outcome in all those with missing data in the comparison arm), and (d) worst-case scenario (i.e. unfavourable outcome in all those with missing data in the intervention arm and favourable outcome in all those with missing data in the comparison arm).

#### Data synthesis

The data obtained will be described in detail. We will pool the data and perform meta-analyses where feasible. In general, this will be feasible for studies utilising the same design and that address similar questions with respect to the population, intervention, comparison (if any) and outcomes. The data from randomised versus non-randomised studies (for long-term safety outcomes) will be analysed separately. Where data cannot be pooled by meta-analysis, we will use the Synthesis Without Meta-analysis (SWiM) guideline checklist [28] to minimise the bias(es) arising from the quantitative narrative synthesis of data. This checklist comprises 9 items encompassing aspects of data synthesis including grouping of studies, methods for synthesising data, presentation of the data and limitations of the synthesis.

#### Statistical analysis

We will present the data with descriptive statistics and provide pooled estimates of outcome parameters (with 95% confidence intervals), wherever meta-analysis is feasible. Outcomes reported through dichotomous variables will be expressed as proportions and compared within and/or between the groups (where applicable) using odds ratios. Outcomes reported through continuous variables will be expressed as mean (SD) and compared within and/or between groups (where applicable) using weighted mean differences. For continuous variables expressed as median (IQR), efforts will be made to convert the values to mean (SD). The default analysis will be the random effects model.

#### Subgroup analysis

Subgroup analyses will be conducted on studies with human participants by (i) age group viz. infants (age < 2 years), children (2–18 years) and adults (> 18 years); (ii) dosage of vaccine (in studies testing different doses using the same microneedle); and (iii) impact of microneedle length (in studies testing the same vaccine delivered through microneedles of different lengths).

#### Sensitivity analysis

Sensitivity analysis will be undertaken to assess the impact of low(er) quality studies on the review findings.

#### Assessment of heterogeneity

Heterogeneity amongst RCTs will be explored by visual inspection of forest plots as well as using the Higgins and Thompson *I*^2^ method [29]. We will interpret heterogeneity as outlined in the *Cochrane Handbook for Systematic Reviews of Interventions*: 75–100%: considerable heterogeneity, 50–90%: may represent substantial heterogeneity, 30–60%: may represent moderate heterogeneity and 0–40%: might not be important [30]. Where *I*^2^ is greater than 50%, we will try to identify possible explanations using subgroup analysis and meta-regression analysis based on the most important characteristics of the studies.

#### Assessment of the methodological quality of included studies

Two authors will independently assess the methodological quality of included studies. Randomised trials will be evaluated using version 2 of the Cochrane Risk-of-Bias Tool (RoB2) for randomised trials [31].

Observational studies will be assessed using the Newcastle–Ottawa Scale [32]. The scale considers three factors viz. (i) selection, including representativeness of the exposed cohort, selection of the non-exposed cohort, ascertainment of exposure and demonstration that at the start of the study the outcome of interest was not present; (ii) comparability, assessed on the basis of study design and analysis, and whether any confounding variables were adjusted for; (iii) outcome, based on the follow-up period and cohort retention, and ascertained by independent blind assessment, record linkage or self-report. The quality of the studies (good, fair and poor) will be rated by awarding stars in each domain following the guidelines of the Newcastle–Ottawa Scale. A ‘good’ quality score will require 3 or 4 stars in selection, 1 or 2 stars in comparability and 2 or 3 stars in outcomes. A ‘fair’ quality score will require 2 stars in selection, 1 or 2 stars in comparability and 2 or 3 stars in outcomes. A ‘poor’-quality score will reflect 0 or 1 star(s) in selection, 0 stars in comparability or 0 or 1 star in outcomes.

For case series and case studies, we will use the Joanna Briggs Institute (JBI) Critical Appraisal Checklist for case series [33].

We will assess the risk of bias across included studies in two ways, as per the Cochrane Handbook guidelines [30]. First, we will assess the risk of bias for an individual outcome, by making judgements about evidence quality. Second, we will try to assess the overall risk of bias across included studies by making judgements on empirical evidence of bias, likely direction of bias, and likely magnitude of bias.

#### Assessment of reporting biases

Wherever possible, we will obtain the original trial protocols for comparison with the published papers to ensure that all outcomes were reported. If this is not possible, we will scrutinise the ‘Methods’ section of publications to ensure full reporting of all measured variables. We will use the Outcome Reporting Bias in Trials (ORBIT) classification system to highlight missing or incomplete outcome reporting of the outcomes [34]. If negative data were not fully reported, we will contact the primary investigators for these data. We will explore reporting bias using a funnel plot. We will also assess publication bias by looking for evidence of conference presentations not followed by subsequent journal publications.

#### Assessment of confidence in the synthesis of findings

Two reviewers will independently assess the quality of evidence of each outcome based on five GRADE considerations, i.e. study limitations, consistency of effect, imprecision, indirectness and publication bias. We will use the methods described in the *Cochrane Handbook for Systematic Reviews of Interventions* [30], employing the GRADEpro GDT software. We will justify decisions to downgrade or upgrade the quality using footnotes with comments. We will also consider the overall quality of evidence across outcomes. The quality of evidence will be rated as high, moderate, low or very low.

## Discussion

Intradermal vaccination provides a potentially efficacious, safe and economic option to administer vaccines to human beings. It has great potential considering that it is the standard approach used for administering millions of doses of the BCG vaccine, as well as the tuberculin skin test. Intradermal administration is also used for allergy tests using the skin prick method. Some vaccines such as the inactivated polio vaccine (IPV) could be administered in a lower dosage through the intradermal route, compared to conventional intramuscular vaccination. Based on these observations, intradermal vaccination using microneedles could be an even safer, far less painful and more acceptable process compared to intradermal vaccination using conventional needles. Preliminary studies in animal models as well as small-scale human studies constitute the limited body of evidence on the subject. To date, there is no well-designed systematic review examining the evidence on efficacy, safety and pain responses in human beings (adults and children) for vaccines administered through the intradermal route using microneedles. This systematic review is planned to address this knowledge gap.

We believe that this is the first comprehensive effort to systematically identify and synthesise evidence on this subject. Some of the strengths of the proposed review are prior publication of the protocol for peer review, literature search through several databases, inclusion of published as well as unpublished studies, inclusion of multiple outcome measures (potentially covering all important methods of presenting data on efficacy and safety) and the robust data analysis plan.

This review also has some limitations. Our search strategy may miss some sources of information available in dissertations, conference presentations and in-house databases. We will consider these limitations when we draw conclusions from this review.

We plan to disseminate the completed systematic review through a peer-reviewed journal publication. Data that cannot be included in the published version will be made available to anyone, on request. We expect the results of the review to be of immense benefit to policy-makers, vaccine manufacturers and of course healthcare professionals delivering vaccinations. It will also be useful to clinical researchers working in the field of vaccine delivery.

## Supplementary Information

**Additional file 1.** Search strategy in PubMed.

## Data Availability

The dataset used or analysed during the proposed systematic review will be available from the corresponding author on reasonable request.

## Abbreviations

BCG: Bacille Calmette-Guerin
IPV: Inactivated polio vaccine
JBI: Joanna Briggs Institute
MeSH: Medical Subject Headings
MN: Microneedle
MR: Measles-Rubella
NOS: Newcastle–Ottawa Scale
ORBIT: Outcome Reporting Bias in Trials
PPD: Purified protein derivative
PRESS: Peer Review of Electronic Search Strategies
PRISMA: Preferred Reporting Items for Systematic Review and Meta-Analysis Protocols
PROSPERO: Prospective Register of Systematic Reviews
RCT: Randomised controlled trials
RSV: Respiratory syncytial virus
SoQF: Summary of Qualitative Findings
SWiM: Systematic Review without Meta-analysis
TT: Tetanus toxoid
