# Supplementary material for: Evaluation of efficacy and safety of intradermal delivery of vaccines through microneedle(s) in human beings: a protocol for a systematic review
Source: Syst Rev. 2022 Aug 13;11:170. doi: 10.1186/s13643-022-02046-8 (PMC9375374; doi:10.1186/s13643-022-02046-8)
Supplement: Supplementary file 1 — Additional file 1. Search strategy in PubMed. [file 13643_2022_2046_MOESM1_ESM.docx]

Supplementary File 1. Search strategy in Pubmed

((("micro"[All Fields] OR "micros"[All Fields]) AND ("needle s"[All Fields] OR "needled"[All Fields] OR "needles"[MeSH Terms] OR "needles"[All Fields] OR "needle"[All Fields] OR "needling"[All Fields] OR "needlings"[All Fields])) OR ("microneedle"[All Fields] OR "microneedles"[All Fields] OR "microneedling"[All Fields])) AND (("intra"[All Fields] AND ("dermalive"[Supplementary Concept] OR "dermalive"[All Fields] OR "dermalive"[All Fields] OR "dermal"[All Fields] OR "dermally"[All Fields])) OR ("intradermal"[All Fields] OR "intradermally"[All Fields] OR "intradermic"[All Fields] OR "intradermically"[All Fields]) OR ("dermalive"[Supplementary Concept] OR "dermalive"[All Fields] OR "dermalive"[All Fields] OR "dermal"[All Fields] OR "dermally"[All Fields]) OR ("dermis"[MeSH Terms] OR "dermis"[All Fields])) AND ("vaccin"[Supplementary Concept] OR "vaccin"[All Fields] OR "vaccination"[MeSH Terms] OR "vaccination"[All Fields] OR "vaccinable"[All Fields] OR "vaccinal"[All Fields] OR "vaccinate"[All Fields] OR "vaccinated"[All Fields] OR "vaccinates"[All Fields] OR "vaccinating"[All Fields] OR "vaccinations"[All Fields] OR "vaccination s"[All Fields] OR "vaccinator"[All Fields] OR "vaccinators"[All Fields] OR "vaccine s"[All Fields] OR "vaccined"[All Fields] OR "vaccines"[MeSH Terms] OR "vaccines"[All Fields] OR "vaccine"[All Fields] OR "vaccins"[All Fields] OR ("vaccin"[Supplementary Concept] OR "vaccin"[All Fields] OR "vaccination"[MeSH Terms] OR "vaccination"[All Fields] OR "vaccinable"[All Fields] OR "vaccinal"[All Fields] OR "vaccinate"[All Fields] OR "vaccinated"[All Fields] OR "vaccinates"[All Fields] OR "vaccinating"[All Fields] OR "vaccinations"[All Fields] OR "vaccination s"[All Fields] OR "vaccinator"[All Fields] OR "vaccinators"[All Fields] OR "vaccine s"[All Fields] OR "vaccined"[All Fields] OR "vaccines"[MeSH Terms] OR "vaccines"[All Fields] OR "vaccine"[All Fields] OR "vaccins"[All Fields]) OR ("immune"[All Fields] OR "immuned"[All Fields] OR "immunes"[All Fields] OR "immunisation"[All Fields] OR "vaccination"[MeSH Terms] OR "vaccination"[All Fields] OR "immunization"[All Fields] OR "immunization"[MeSH Terms] OR "immunisations"[All Fields] OR "immunizations"[All Fields] OR "immunise"[All Fields] OR "immunised"[All Fields] OR "immuniser"[All Fields] OR "immunisers"[All Fields] OR "immunising"[All Fields] OR "immunities"[All Fields] OR "immunity"[MeSH Terms] OR "immunity"[All Fields] OR "immunization s"[All Fields] OR "immunize"[All Fields] OR "immunized"[All Fields] OR "immunizer"[All Fields] OR "immunizers"[All Fields] OR "immunizes"[All Fields] OR "immunizing"[All Fields]))
